# Regulating Emotional Responses to Climate Change – A Construal Level Perspective

**DOI:** 10.3389/fpsyg.2018.00629

**Published:** 2018-05-01

**Authors:** Emma Ejelöv, André Hansla, Magnus Bergquist, Andreas Nilsson

**Affiliations:** Department of Psychology, University of Gothenburg, Gothenburg, Sweden

**Keywords:** Construal Level Theory, spatial distance, climate change, risk communication, emotions, emotion-regulation strategies

## Abstract

This experimental study (*N* = 139) examines the role of emotions in climate change risk communication. Drawing on Construal Level Theory, we tested how abstract vs. concrete descriptions of climate threat affect basic and self-conscious emotions and three emotion regulation strategies: changing oneself, repairing the situation and distancing oneself. In a 2 × 2 between subjects factorial design, climate change consequences were described as concrete/abstract and depicted as spatially proximate/distant. Results showed that, as hypothesized, increased self-conscious emotions mediate overall positive effects of abstract description on self-change and repair attempts. Unexpectedly and independent of any emotional process, a concrete description of a spatially distant consequence is shown to directly increase self-change and repair attempts, while it has no such effects when the consequence is spatially proximate. “Concretizing the remote” might refer to a potentially effective strategy for overcoming spatial distance barriers and motivating mitigating behavior.

## Introduction

There is scientific consensus that anthropogenic climate change is an urgent threat to our planet. Nevertheless, public perceptions remain ambivalent and people continuously fail to take necessary measures to mitigate the negative consequences (O’Neill and Whitmarsh, 2009). In order to reach the goal set out by the UN of limiting mean temperature rise to 2°C (United Nations, 2015), both the global and immediate nature of climate change must be communicated in a way that is personally engaging and motivates behavior change. But how do we communicate a risk that most people simultaneously perceive as both alarmingly present and yet elusively distant? Climate change risk communication is often based on the notion that making climate change appear more proximal in space and time would increase risk perception, involvement, and mitigating actions (Jones et al., 2016). However, recent research has suggested that simply communicating risks of climate change as geographically closer might not in itself increase such actions. Rather, it seems to alter what information guides decision-making (Brügger et al., 2016). Some risks may in fact also be unique to or more severe at spatially distant places and, thus, could not easily be brought to feel closer. New effective ways of communicating climate threat are therefore much called for.

In the present research, we aim to compare different types of risk communication messages, and suggest that emotion mediates readiness to act against climate change threat, succeeding different descriptions of this threat. Using Construal Level Theory (Liberman and Trope, 2003; Trope and Liberman, 2010) as theoretical framework, an experiment was conducted to test whether and how varying the abstractness of descriptions of climate change consequences, e.g., describing consequences as a heat wave or increase in mean temperature, influence emotional responses and motivation to mitigating action across short and long spatial distances to these consequences. This approach could increase our understanding of what makes risk communication and policy making successful.

### Construal Level Theory and Climate Change

Construal Level Theory (CLT) states that objects, events, and constructs (e.g., consequences of climate change) can be thought of in more or less abstract terms depending on the psychological distance to them. In brief, the further away something is perceived to be from ones immediate experience, the more abstract the construct or event will be perceived as, i.e., we say that the *construal* of the event is abstract. Four types of psychological distances have been proposed: temporal, social, hypothetical, and spatial (Trope and Liberman, 2010), of which the latter is focused on in the present research. Specifically, an event that takes place geographically far away should make us perceive and process it in a more abstract and general way, i.e., the event is construed at a high level. Conversely, an event that is geographically close to us should give rise to a concrete and context-dependent (i.e., low-level) construal (e.g., Bar-Anan et al., 2006; Liberman and Trope, 2008). For example, when people think about an event in an abstract way, they typically also perceive it to be further away geographically than it actually is. Thinking abstractly also makes people susceptible to influences of other abstract, high-level, information, such as values and attitudes (e.g., Eyal et al., 2009; Ledgerwood et al., 2010).

Now consider how ones’ construal level would be affected when reading a climate-change risk communication message that relatively concretely describes spatially distant consequences and how these unfolded (e.g., how hurricane Katrina affected New Orleans from a Europeans’ perspective). Would it evoke a low-level construal – driven by a dominant effect of a concrete description - or a high-level construal – driven by a dominant effect of spatial distance? Or could there perhaps be counteracting effects of concrete description and spatial distance that evoke an intermediate construal level? It seems that previous research on CLT offers no up-front answers to these questions. Hence, when applying CLT in communicating risks about climate change, we have considered two possible ways in which spatial (psychological) distance to consequences and the description of those consequences (abstract vs. concrete) can affect a persons’ construal level.

### Two Construal Level Models

The first is the additive model, in which spatial distance and description abstractness each can have an independent effect and contribute to a high or low construal level (see e.g., Bar-Anan et al., 2006; Henderson et al., 2006; Williams et al., 2014). Accordingly, an abstract description of a spatially distant consequence, – i.e., when psychological distance and description abstractness are fitted – should yield the highest (most abstract) construal level, while a concrete description of a spatially proximate consequence, also representing a fitted description, should yield the lowest (most concrete) construal level. Mismatched descriptions (e.g., concrete description of spatially distant consequence) would on the other hand imply an intermediate construal level in the additive model^1^.

The second way spatial distance and description abstractness could affect construal level is by interaction. An interaction model would predict that spatial distance and description abstractness exert conditional or multiplicative effects on construal level. Such a model would be supported if for instance describing a spatially distant consequence as concrete evokes a low-level construal, while at the same time describing a spatially proximate consequence as abstract does not evoke a high-level construal. To our knowledge, no previous study has investigated this type of interaction model (but see Rabinovich et al., 2009 for a similar line of reasoning), which is why we treat the additive model as more plausible while the interaction model is as a theoretical possibility worth exploring.

### Emotional Response to Psychological Distance

Risk communication on climate change has increasingly focused on emotion and related emotion-regulation strategies (Roeser, 2012; Panno et al., 2015) and psychological distance to climate change consequences has also been shown to affect the intensity with which emotions are experienced (Van Boven et al., 2010; McDonald et al., 2015). Emotions are generally felt less intense with increased psychological distance to the emotion-eliciting event. Conversely, when people experience intense emotions, they typically perceive the emotion-eliciting event to be psychologically proximate (e.g., Van Boven et al., 2010; Williams et al., 2014). However, whether emotional intensity is increased by psychological proximity, may depend on what type of emotion is experienced. For example, although basic emotions like fear and anger may involve proximal threats (Giner-Sorolla, 2001), self-conscious emotions like shame or guilt seem to require taking a psychologically (in this case socially) distant perspective, seeing oneself from another person’s perspective and judging whether ones’ actions are appropriate or not (Agerström et al., 2012). We propose that a high construal level of climate change consequences may evoke stronger self-conscious and weaker basic emotions than a low construal level.

Distinguishing self-conscious from basic emotions is also important as the former are more strongly related to acting on long-term goals (Carver and Scheier, 1990; Baumeister et al., 2010) while the latter are more strongly associated with working toward short-term goals (Frijda et al., 1989; Tracy and Robins, 2004). Self-conscious emotions can thus prove especially important for climate change risk communication, as it enables people to act on the long-term goal of combating negative consequences of climate change. There is also previous evidence suggesting that basic and self-conscious emotions are regulated differently.

### Regulating Basic and Self-Conscious Emotions

Emotional regulation concerns the strategies people use to influence what they are feeling, how intensely they are feeling it and when they are feeling it (Gross and John, 2003). Basic emotions have been linked to regulation strategies like distancing from climate change threat (Lorenzoni et al., 2006; O’Neill and Nicholson-Cole, 2009; Schoenefeld and McCauley, 2015; McDonald et al., 2015), which would be advantageous for the individual but disadvantageous for the environment. Self-conscious emotions on the other hand are not as likely to be regulated by distancing, as the experience of these emotions requires taking a psychologically distant perspective. To increase that distance further would thus only increase the intensity of the negative self-conscious emotion (Katzir and Eyal, 2013). Self-conscious emotions have instead been found to lead to regulation strategies such as motivation to repair a situation or change oneself (Lickel et al., 2014), which would be advantageous for both the individual and the environment. In this study, we will look at the two environmentally beneficial emotion-regulation strategies (changing oneself for the environment and repairing the situation) and one environmentally harmful strategy (distancing from climate change) identified by Lickel et al. (2014).

### The Present Study

The aim of the present study is to examine emotional reactions to and related emotion-regulation strategies following different descriptions of climate change consequences varying in spatial distance and description abstractness. We will test the additive and interaction models and in each treat self-conscious and basic emotions as mediating variables and emotion-regulation strategy as dependent variable. In line with the more established additive model, we expected that a spatially distant and an abstract consequence each would (i) indirectly increase regulation attempts of self-change and repair via strengthening self-conscious emotions (Hypotheses 1) and (ii) indirectly decrease regulation attempts of distancing via decreasing basic emotions (Hypotheses 2).

## Materials and Methods

### Participants and Procedure

One hundred and thirty nine individuals (Age *M* = 29.8, *SD* = 10.1, 64.7% females), recruited from a participant pool at the University of Gothenburg, Sweden, completed an online survey, without receiving monetary compensation. Participants were randomly assigned in a 2 (spatial distance: Proximal vs. Distal) × 2 (description: Concrete vs. Abstract) between-groups design, using Qualtrics Survey Software. Each participant read one of four scenarios describing consequences of climate change and then watched a short video clip depicting a severe rainstorm in a suburban area. In the spatially close condition, participants read that the storm affected Sweden, while participants in the distal condition read that the storm affected Canada (a geographically distant country, but comparable in terms of climate zone and geo-political situation). Consequence description was framed as concrete by describing tangible consequences of climate change (e.g., heat waves and flooding) that would affect Sweden/Canada or abstract by describing intangible consequences of climate change (e.g., an increase in mean global temperature and a rise of mean sea-level) that would affect Sweden/Canada. Moreover, in the concrete versus abstract scenario descriptions respondents were asked to think about *how* (for concrete) versus *why* (for abstract) climate change consequences will affect Sweden/Canada. The use of *how* (making respondents think about the specific process of climate change impact) and *why* (making respondents think about the cause of climate change impact) questions is a common practice when priming a low and high-level construal, respectively (e.g., Freitas et al., 2004; Liberman and Trope, 2008). After reading the scenario description and watching the video, participants were asked to think about the previously mentioned climate change consequences and then answered questions about how intensely they experienced 15 different emotions (shame, guilt, embarrassment, pride, anger toward oneself, anger toward others, sadness, fear, disappointment, worry, helplessness, interest, joy, relief, hope) presented in random order (*1 - not at all intense* to *9* – *very intense*) and the eleven emotion-regulation questions (see Lickel et al., 2014 for complete list of items) measuring self-change, repair or distancing (also presented in random order). The emotion-regulation questions were modified to specifically attain to the question of climate change. For example, self-change was measured by items like “*I feel the need to change myself when I think about climate change*,” repair was measured by items like “*I feel that I should apologize for my own impact on climate change*” and distancing by items like “*I want to distance myself from the issue of climate change as much as possible*,” all measured on a 1–9 Likert scale (*1 – strongly disagree* to *9 – strongly agree*). Finally, as a manipulation check, participants were asked to rate the statement “*I felt like the storm [in the video-clip] was happening far away from Me.*” on a 1–9 Likert scale (*1 – strongly disagree* to *9* – *strongly agree*).

#### Manipulation Check

To check whether participants in the spatially distant condition perceived the storm to be further away and participants in the spatially close condition perceived it to be closer, we performed a one-way ANOVA, with spatial distance as independent variable and spatial perception as dependent variable. The difference between Sweden (*M* = 3.34, *SD* = 2.24) and Canada (*M* = 3.52, *SD* = 2.55) was in the expected direction but lacked statistical significance, *F*(1,136) = 0.19, *p* = 0.67. Note that the manipulation check only pertains to how distant the storm in the video-clip was perceived, not the separate consequence descriptions that either affected Sweden or Canada. It is thus possible that spatial distance was manipulated at both earlier and later stages only in the text scenario descriptions, which our question did not check for.

### Measures

First, a scale for self-conscious emotions was created (cf. Tracy and Robins, 2004; Agerström et al., 2012), containing emotions of shame, guilt, embarrassment, anger toward oneself and pride. The scores for pride were inverted before entered into the scale, due to it being a positive self-conscious emotion. The scale had acceptable internal reliability (Cronbach’s α = 0.77; see Nunnally, 1978). Second, a scale for basic emotions was constructed (cf. Arnold, 1960; Frijda, 1986), containing the emotions sadness, anger toward other, fear, disappointment, worry, helplessness, relief, joy and interest. The scores for positive emotions were inverted. The scale showed acceptable internal reliability (Cronbach’s α = 0.78). Finally, the scales for motivation to self-change, repair and distancing (Lickel et al., 2014) were tested for internal reliability. The scale for repair showed lower reliability, (Cronbach’s α = 0.66, 3 items), while the scale for self-change (Cronbach’s α = 0.86, 4 items) distancing both showed acceptable reliability (Cronbach’s α = 0.76, 4 items).

## Results

### Test of Additive Model

**FIGURE 1:**
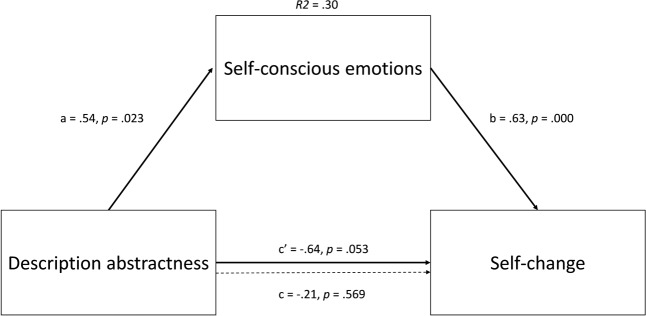
Direct and indirect effects in model 4 when spatial distance is entered as covariate. Dotted line denotes the effect of description abstractness on self-change when self-conscious emotion is not included as a mediator.

**FIGURE 2:**
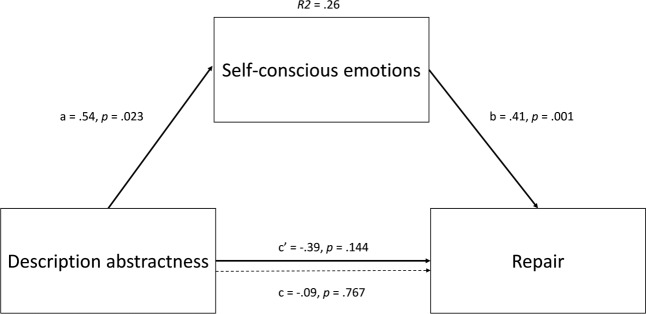
Direct and indirect effects in model 4 when spatial distance is entered as covariate. Dotted line denotes the effect of description abstractness on repair when self-conscious emotion is not included as a mediator.

To firstly test the additive model (and our hypotheses), we conducted six simple mediation analyses (PROCESS v.2.16.3, model 4, Hayes, 2013 – see **Figures 1**, **2**), two for each of the three emotion-regulation strategies. In the first set of analyses, description abstractness was entered as independent variable (concrete = 0 vs. abstract = 1), spatial distance as covariate (Sweden = 0 vs. Canada = 1), self-conscious and basic emotions as mediators and emotion-regulation strategy as dependent variable. The second set of analyses was identical to the first, except that spatial distance was entered as the independent variable and description abstractness as the covariate. All confidence intervals (BCCI) reported were bias corrected and bootstrapped from 5000 samples and are at a 95% confidence level.

Partially supporting H1, abstractly described consequences, when entered as independent variable, positively influenced both willingness to self-change, *ab* = 0.34, BCCI [0.06, 0.71], and repair, *ab* = 0.22, BCCI [0.05, 0.46], via a positive influence on self-conscious emotions, β = 0.54, *p* = 0.02, *d* = 0.39. Partial standardization of these indirect effects reveal that abstract consequence description (via its influence on self-conscious emotion) increased willingness to self-change and repair by 0.15 and 0.12 standard deviations, respectively (which may exemplify small effects). Yet, in contrast to H1, spatial distance, when entered as independent variable, did not influence self-conscious emotion (β = 0.04, *p* = 0.87, *d* = 0.03). The R^2^ for the total effect model was 0.009 for self-change and 0.001 for repair. Refuting H2, basic emotions were influenced neither by description abstractness (β = 0.26, *p* = 0.27, *d* = 0.19) nor by spatial distance (β = 0.09, *p* = 0.70, *d* = 0.06) and were unrelated to distancing attempts (β = 0.03, *p* = 0.78, *r* = 0.02).

### Test of Interaction Model

**FIGURE 3:**
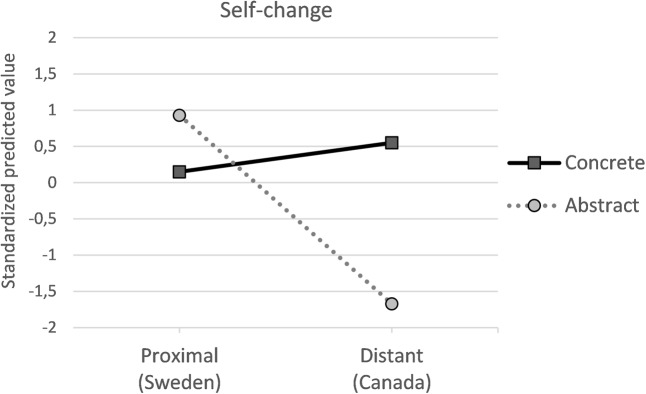
Standardized predicted mean values for willingness to self-change.

**FIGURE 4:**
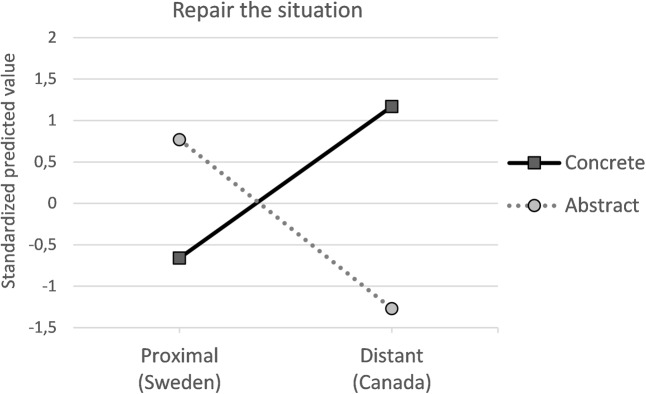
Standardized predicted mean values for willingness to repair.

To allow testing of the interaction model we conducted six moderated mediation analyses (model 8 – see **Figures 3**, **4**), two for each of the three emotion-regulation strategies. As when testing the additive model, we entered description abstractness as independent variable and spatial distance as moderator in the first set of analyses. Analyses revealed that when consequences occurred in Canada, the concrete description directly increased self-change, *b* = -1.2, *p* = 0.01, *d* = 0.44, BCCI [-2.1, -0.28] and repair *b* = -0.86, *p* = 0.02, *d* = 0.39, BCCI [-1.61, -0.11], compared with the abstract description (see **Figure 4**). However, when consequences occurred in Sweden, the concrete description influenced neither self-change: *b* = -0.11, *p* = 0.81, *d* = 0.04, BCCI [-1.0, 0.77] nor repair: *b* = 0.05, *p* = 0.90, *d* = 0.02, BCCI [-0.68, 0.78].

In the second set of analyses, spatial distance was entered as independent variable and description abstractness as moderator. When abstract consequences were described, the reference to Canada decreased self-change, *b* = -0.94, *p* = 0.04, *d* = 0.36, BCCI [-1.8, -0.05] and tended to decrease repair, *b* = -0.52, *p* = 0.16, *d* = 0.24, BCCI [-1.25, 0.21]. When concrete consequences were described the reference to spatial distance had no effect, influencing neither self-change: *b* = 0.14, *p* = 0.75, *d* = 0.05, BCCI [-0.75, 1.04] nor repair: *b* = 0.39, *p* = 0.30, *d* = 0.18, BCCI [-0.35, 1.13]). However, spatial distance and description abstractness did not interact to affect self-conscious (*p* = 0.89, index of moderated mediation, self-change = 0.04, BCCI [-0.49, 0.65], repair = 0.02, BCCI [-0.33, 0.46]) or basic emotions (*p* = 0.54, index of moderated mediation, self-change = 0.1, BCCI [-0.18, 0.6], repair = 0.1, BCCI [-0.18, 0.52]). These results indicate that description abstractness and spatial distance exert multiplicative effects on emotion-regulation strategies only directly.

## Discussion

This research aimed to investigate if and how risk communication messages building on different descriptions of climate change consequences affect our emotional response and emotion-regulation strategies. We found support for our hypothesis that describing climate change consequences in a more abstract way elicits more self-conscious emotions than describing them more concretely. Self-conscious emotions further mediated influences of description abstractness on adaptive responses to climate change such as willingness to self-change and repair. Unexpectedly, we corroborated no such emotional or mediational effects of spatial distance. Yet, spatial distance was for that matter not unimportant but was seen to play a different role. We found that only when consequences were spatially distant did concretizing (vs. abstracting) their description exert an effect, directly positively influencing regulation attempts of willingness to self-change and repair. When consequences were instead described as occurring nearby in space, abstracting (vs. concretizing) did not matter, however, leaving the regulation attempts unaffected. As a possible theoretical implication and practical application (in facilitating mitigating action), this finding would suggest that it is easier to offset spatially distant consequences via concretization – bringing them psychologically closer or lowering a high-level construal – than to offset spatially proximate consequences via abstraction – bringing them into psychological distance or raising a low-level construal.

Furthermore, there was no indication that a concrete representation of climate change consequences would elicit basic emotions or that experiencing intense basic emotions would lead to willingness to distance oneself from climate change. However, the fact that distancing was not predicted by either type of emotional intensity is, perhaps, unsurprising given that the process of distancing from an unpleasant event involves reducing ones’ emotional response (Ayduk and Kross, 2010). Distancing from climate change in particular has been shown to be related to experiencing a lower degree of negative emotions (Homburg et al., 2007; Ojala, 2013). If people were distancing themselves the process presumably began already while reading the scenario-descriptions and watching the video-clip, which might have resulted in a lower rated emotional intensity for those participants. It should be noted that the ratings for distancing were relatively low, compared to self-change and repair. The items measuring distancing could also have been too blunt to capture this construct, as admitting to distancing from an important issue like climate change might not be socially desirable. As the measure for distancing might not have accurately captured the construct, we cannot comment as to whether a high intensity of basic emotions predicts distancing or not. This construct might be better measured implicitly rather than, as in this case, explicitly.

That people experience and are guided by high-level constructs like self-conscious emotions when climate change consequences are mentally represented as abstract is a new finding in the field of climate change communication, but is in line with previous research on decision-making (e.g., Eyal et al., 2009; Ledgerwood et al., 2010). This shows that the emotional effects of psychological distance previously established in other research domains are also relevant for research on environmental risk communication. Interestingly, when consequences are represented as concrete, people do, however, not seem to be guided by basic emotions like fear and sadness. Self-conscious emotions are often experienced simultaneously with basic emotions, but some situations elicit only basic emotions as they are not contingent on a self-evaluative process (Beer and Keltner, 2004). That basic emotions were experienced equally strong in both the concrete and abstract frame might thus not be surprising considering that what is unique about the concrete frame is not the presence of basic emotions but rather the absence of self-conscious emotions. In other words, the concrete frame elicits only basic emotion while the abstract frame elicits both basic and self-conscious emotions. What guides mitigating motivation when people are in a concrete mind-set might thus be something other than emotion. Additional research is certainly needed here.

That spatial distance did not affect emotional intensity is in contrast to previous research where increased psychological distance generally minimizes emotional experience (e.g., Van Boven et al., 2010; Williams et al., 2014). There are three possible explanations for this. Firstly, the fact that we varied spatial distance by comparing two countries that are highly similar with respect to their geo-political situation, to deliberately try to keep social distance – that is, how (dis)similar other people are to us – constant, could have acted to reduce emotional response. It is possible that the emotional effect of spatial distance demonstrated in previous research may have been exaggerated by not controlling for this potential confound. Secondly, consequence description could have been a more direct manipulation of construal level, making spatial distance redundant. Lastly, description abstractness and spatial distance might not have had the same impact on construal level, as description abstractness was manipulated both by describing abstract (vs. concrete) consequences and by asking participants to think about *why* (vs. *how*) consequences were occurring. Spatial distance on the other hand was only manipulated by the wording of Sweden or Canada. This could partly explain why only description abstractness had an impact on emotional response.

There was furthermore no overall direct effect of spatial distance on mitigating motivation, lending support to previous studies (e.g., Schoenefeld and McCauley, 2015; Brügger et al., 2016). However, our findings point to a complex interactive effect of spatial distance in that only when consequences were abstractly described did spatial distance demotivate willingness to self-change and repair. According to Rabinovich et al. (2009), presenting something as simultaneously concrete and distant or abstract and proximate (i.e., non-fitted descriptions) allows people to think both about concrete steps to take in order to mitigate a problem (cf. *how*) whilst also think about the importance of mitigating the problem (cf. *why*). Thus, when we think about climate change consequences as both abstract and distant problems, it could communicate the significance of climate change but fail to motivate self-change and repair because it doesn’t get people thinking about *how* to take action. Making a spatially distant problem more concrete may then have a positive effect because it informs people how to respond. Conversely, when we think about climate change consequences as spatially proximate and concrete problems, we might recognize action alternatives but fail to recognize the importance of action. That making a spatially proximate problem more abstract did not encourage action may (or may not) support this interaction account. The possibility of a positive effect of non-fitted descriptions was at most hinted at and future research is certainly needed to further explore this possibility. The scale for repair further showed lower reliability (Cronbach’s α = 0.66) than the scale for self-change and distancing, which might help explain why the interaction effect of spatial distance and description abstractness was more pronounced for self-change. Future studies might consider using scales that have already been adapted to the climate change context.

In the debate about whether to communicate risks of climate change as spatially distant or proximate, a discussion of consequence description abstractness is missing. Even though construal level is often inferred from psychological distance and vice versa, perceiving climate change consequences as spatially distant must not lead to the same effect as perceiving them as abstract. In fact, our findings suggest that concretizing description of a spatially distant consequence may bring it as psychologically close as a spatially proximate consequence, promoting a similar level of mitigating motivation. This study contributes to this debate by highlighting that there can be a negative effect of perceiving climate change consequences as spatially distant, but only when they are simultaneously described in an abstract way. Spatial distance to risk might thus not in itself reduce willingness to act on climate change, but rather be dependent on other factors (such as consequence information) that might affect level of construal. This provides an indication that there is no “one-way”-strategy to best communicate the risks of climate change. Depending on which aspects one wishes to communicate different framings might be warranted. When environmental risks are occurring at a distance, they should be communicated in a concrete and tangible manner to motivate mitigating action. If, however, risks are close to home, an abstract representation of it could indirectly lead to self-change, via self-conscious emotions.

## Conclusion

This study contributes to our understanding of how risk communication can be designed to mitigate negative consequences of climate change. In risk communication we highlight both the importance of construal level and the mediating role of self-conscious emotions. Climate change risk communication might thus make an effort to induce this type of self-relevant emotion rather than relying on basic emotions, like fear, in order to promote sustainable behavior. While abstract representations lead to mitigating motivation by increasing self-conscious emotions, future research should consider more specifically which factors are involved in the concrete representation of climate change’s ability to motivate self-change. Self-conscious emotions are often related to long-term goals, and if this makes also abstract representations of risks better suited for long-term action is a path worth investigating. The results from this study are further in line with that of Brügger et al. (2016) in that relying on making climate change appear proximate is not a clear cut strategy for increasing mitigating behavior motivation. Rather, describing high- and low-level aspects of climate change simultaneously may help us reconcile the discrepancy between an abstract problem and concrete solution.

## Ethics Statement

This study did not warrant ethical vetting, as it did not record any sensitive information about the research participants or involved sensitive populations. Informed consent was given by the participants before taking part in the online experiment.

## Author Contributions

All authors listed have made a substantial, direct and intellectual contribution to the work, and approved it for publication.

## Conflict of Interest Statement

The authors declare that the research was conducted in the absence of any commercial or financial relationships that could be construed as a potential conflict of interest.

## Footnotes

1 Note that, compared to spatial distance, description abstractness could still be assumed to have a larger additive effect on construal level because it would be a more direct and powerful manipulation of construal level.
